# Diagnostic Accuracy of an At-Home, Rapid Self-test for Influenza: Prospective Comparative Accuracy Study

**DOI:** 10.2196/28268

**Published:** 2022-02-22

**Authors:** Rachel E Geyer, Jack Henry Kotnik, Victoria Lyon, Elisabeth Brandstetter, Monica Zigman Suchsland, Peter D Han, Chelsey Graham, Misja Ilcisin, Ashley E Kim, Helen Y Chu, Deborah A Nickerson, Lea M Starita, Trevor Bedford, Barry Lutz, Matthew J Thompson

**Affiliations:** 1 Department of Family Medicine University of Washington Seattle, WA United States; 2 Department of Bioengineering University of Washington Seattle, WA United States; 3 Department of Medicine University of Washington Seattle, WA United States; 4 Brotman Baty Institute University of Washington Seattle, WA United States; 5 Department of Genome Sciences University of Washington Seattle, WA United States; 6 Massachusetts Institute of Technology Cambridge, MA United States; 7 Vaccine and Infectious Disease Division Fred Hutchinson Cancer Research Center Seattle, WA United States

**Keywords:** influenza, influenza, rapid testing, acute respiratory illness, self-collection, self-testing, mHealth, mobile health, home collection, home testing, mobile phone

## Abstract

**Background:**

Rapid diagnostic tests (RDTs) for influenza used by individuals at home could potentially expand access to testing and reduce the impact of influenza on health systems. Improving access to testing could lead to earlier diagnosis following symptom onset, allowing more rapid interventions for those who test positive, including behavioral changes to minimize spread. However, the accuracy of RDTs for influenza has not been determined in self-testing populations.

**Objective:**

This study aims to assess the accuracy of an influenza RDT conducted at home by lay users with acute respiratory illness compared with that of a self-collected sample by the same individual mailed to a laboratory for reference testing.

**Methods:**

We conducted a comparative accuracy study of an at-home influenza RDT (Ellume) in a convenience sample of individuals experiencing acute respiratory illness symptoms. Participants were enrolled in February and March 2020 from the Greater Seattle region in Washington, United States. Participants were mailed the influenza RDT and reference sample collection materials, which they completed and returned for quantitative reverse-transcription polymerase chain reaction influenza testing in a central laboratory. We explored the impact of age, influenza type, duration, and severity of symptoms on RDT accuracy and on cycle threshold for influenza virus and ribonuclease P, a marker of human DNA.

**Results:**

A total of 605 participants completed all study steps and were included in our analysis, of whom 87 (14.4%) tested positive for influenza by quantitative reverse-transcription polymerase chain reaction (70/87, 80% for influenza A and 17/87, 20% for influenza B). The overall sensitivity and specificity of the RDT compared with the reference test were 61% (95% CI 50%-71%) and 95% (95% CI 93%-97%), respectively. Among individuals with symptom onset ≤72 hours, sensitivity was 63% (95% CI 48%-76%) and specificity was 94% (95% CI 91%-97%), whereas, for those with duration >72 hours, sensitivity and specificity were 58% (95% CI 41%-74%) and 96% (95% CI 93%-98%), respectively. Viral load on reference swabs was negatively correlated with symptom onset, and quantities of the endogenous marker gene ribonuclease P did not differ among reference standard positive and negative groups, age groups, or influenza subtypes. The RDT did not have higher sensitivity or specificity among those who reported more severe illnesses.

**Conclusions:**

The sensitivity and specificity of the self-test were comparable with those of influenza RDTs used in clinical settings. False-negative self-test results were more common when the test was used after 72 hours of symptom onset but were not related to inadequate swab collection or severity of illness. Therefore, the deployment of home tests may provide a valuable tool to support the management of influenza and other respiratory infections.

## Introduction

### Background

In the most recent influenza season in the United States (October 2019 to April 2020), an estimated 39 to 62 million people were infected, resulting in 18 to 26 million health care visits and 24,000 to 62,000 deaths [1]. The economic impacts are proportional—the 2018 seasonal influenza cost the United States an estimated US $11.2 billion, including US $3.2 billion in direct medical costs and an estimated 20.1 million productive hours lost [2]. Negative impacts on health and the economy may be improved by early interventions to diagnose those with influenza and intervene with antiviral treatment or behavioral changes to reduce transmission.

Diagnosis of influenza based on clinical features alone is inaccurate; therefore, several clinical guidelines support laboratory testing of respiratory tract specimens (usually nasal or nasopharyngeal) to detect the influenza virus. Increasingly, laboratory testing for influenza has shifted to in-clinic testing using point-of-care (POC) devices [3]. Rapid diagnostic tests (RDTs) are a class of POC tests that can be performed with a few simple steps and typically do not require instrumentation or special supplies, raising the possibility for untrained individuals to use these tests outside of clinical settings [4]. Influenza RDTs for home use could potentially expand access to testing and lower costs, thus facilitating earlier diagnosis and reducing the time from symptom onset to appropriate care, such as receiving antiviral treatment or making behavioral changes to minimize spread [5]. The advantages of home testing for influenza and other respiratory viruses could be even more critical in pandemic situations, where isolating cases and limiting contact with potential cases are essential components of containing outbreaks [6,7].

Several studies have already investigated the accuracy of self-swabbing and self-testing for influenza. A recent systematic review of 13 studies found that influenza was detected by self-collected nasal or midturbinate samples, with similar accuracy to samples collected by health care professionals [8]. RDTs tested in routine health care settings have shown sensitivities and specificities of 60% to 70% and 90% to 100%, respectively [9,10]; however, owing to the novelty of home testing, few RDTs have been studied in the home environment. There are currently no Food and Drug Administration tests approved for the detection of influenza at home.

A primary hurdle to at-home testing for influenza or other respiratory viruses is that RDTs are typically less accurate than laboratory-based assays, even when used by health care workers [11,12]. Numerous variables affect the performance of the test, including quality of the sample, infection prevalence, timely testing after illness onset, and lower viral load in less severe cases. These variables have not been well-studied in POC settings [9,13-15] or in at-home populations; to our knowledge, only 1 publicly available study has attempted to assess the accuracy and feasibility of performing an entire self-test at home using an RDT [16].

### Objective

In this study, we assess the accuracy of an influenza RDT conducted at home by lay users with influenza-like-illness compared with that of a self-collected sample by the same individual mailed to a laboratory for reference testing.

## Methods

### Study Design

We conducted a prospective, comparative accuracy study of an at-home influenza RDT in a convenience sample of individuals experiencing acute respiratory illness (ARI). The study was conducted as a substudy within the Seattle Flu Study (SFS), which has conducted city-wide community surveillance for influenza and other respiratory viral infections. The SFS involved same-day self-swab samples [17]; participants who qualified and enrolled in the self-test substudy reported here received an additional at-home influenza RDT. The RDT results were compared with the results of a self-collected midturbinate nasal swab sample returned by mail and tested by a laboratory quantitative reverse-transcription polymerase chain reaction (qRT-PCR) assay as described below [17]. Participants also answered a questionnaire that included information about their symptoms, risk factors, and demographics.

### Ethical Approval

The study was approved by the University of Washington Human Subjects Division (STUDY00006181) and informed consent was obtained prior to study enrollment. Reporting of this study adheres to STARD (Standards for Reporting of Diagnostic Accuracy Studies) guidance [18].

### Recruitment

Participants were recruited using targeted web-based advertisements on websites, including Facebook, Instagram, Twitter, and Google. Additional recruitment occurred through in-person referrals from community kiosks set up by the SFS, health care providers, travel clinics, immigrant and refugee health screenings, local schools, and workplaces. Potential participants were directed to a study website, where they were screened for eligibility based on age, zip code, symptoms, time from symptom onset, and their smartphone operating system. Participants were compensated with US $20 in the form of an electronic gift card for completing the study procedures; if they returned the reference swab but did not complete the surveys, they were still compensated with US $15.

### Sample Size

The recruitment goal was to enroll 3000 participants into the study, assuming 2400 (80%) participants would complete all steps and an influenza prevalence rate of 12.5%, which would have provided us with 300 influenza-positive samples. Following the study’s completion, electronic gift cards of up to US $20 were sent to all participants.

### Participants

Participants were enrolled from February 19 to March 9, 2020, from the Greater Seattle area of Washington, United States, which has a population of 744,000. Eligibility criteria included those who self-identified as having a cough or at least two new or worsening ARI symptoms (ie, feeling feverish, headache, chills or shivering, sore throat, nausea or vomiting, runny or stuffy nose, malaise, muscle or body aches, trouble breathing, diarrhea, rash, ear pain, or discharge) in the previous 72 hours [19,20]. In addition, participants were required to be aged ≥5 years, residing or working within a list of eligible zip codes, able to understand the study instructions in English, and able to use the study app on a Bluetooth-enabled device to conduct study procedures and the Ellume Home Flu Test (EHFT).

### Pretest Data Collection

The participants consented electronically; a parent or legal guardian consented for participants aged <18 years, and assent forms were provided for participants aged 13 to 18 years using REDCap (Research Electronic Data Capture; Vanderbilt University) [21], hosted at the University of Washington Institute of Translational Health Sciences. After consenting, the REDCap instrument obtained the participant’s home address and contact information to allow the delivery of an influenza kit through courier.

### Influenza Kit Components and Delivery

Influenza kits were fabricated by the study team to comply with US regulations for shipping biological substances (Category B) [22]. The influenza kit contained 1 instructional quick start guide; an influenza RDT labeled as a research device (EHFT), which included a midturbinate swab, buffer fluid, dropper, and Bluetooth-enabled sample analyzer; and a reference sample kit containing 1 midturbinate swab (Copan, FLOQSwabs 56380CS01), 1 tube with 3 mL of the viral transport medium (VTM; catalog #220220; Becton, Dickinson and Company Ltd), 1 specimen transport bag with absorbent sleeve (cat. #11215-684; VWR International LLC), 1 return box (S-16524; ULINE), and 1 return mailer (S-3355; ULINE) overpack. If participants reported errors with the EHFT, a second kit was sent out as soon as possible during staffing hours (within 12 hours).

Influenza kits were sent to the participants’ homes within 24 hours of enrollment, with most sent within 2 hours. Enrollments received after business hours were processed and mailed the following morning. Each kit included a unique barcode number (located at the following three places in each kit: on the 3 mL tube, return mailer, and quick start guide) to link surveys, EHFT results, and reference test results for analysis. Participants were asked to confirm that the barcode on their kit’s tube matched that on the quick start guide and enter this barcode in the REDCap survey. In cases where multiple kits were sent to the same household, barcodes entered in REDCap allowed reference samples received in the laboratory to be linked to the correct participants, even when kits were switched between participants. Once participants completed their at-home study procedures, they were instructed to mail their reference sample, using the materials provided, to the University of Washington research laboratory via the US Postal Service within 24 hours of completing their test.

### At-Home Test and Data Collection

Upon receiving their influenza kit at home, participants were instructed to complete a questionnaire on REDCap (sent via email). This included questions about their symptoms and exposure risks, including housing, health conditions, recent travel, and demographics (Multimedia Appendix 1).

Participants were instructed to download the EHFT app onto their Bluetooth-enabled device. The app provided an instructional video, followed by step-by-step on-screen instructions for sample self-collection using the included custom midturbinate swab. Participants were instructed to insert and rotate the swab 3 times around their nasal cavities on both nostrils. They then placed the swab sample in the buffer, added this buffer fluid to the analyzer, and waited for 12 minutes to process the sample. The analyzer then sent test results directly to the EHFT app on the user’s device via Bluetooth and a secure research database. As participants were using an experimental research device, they were blinded to the EHFT test results. Participants received a *thank you* screen in the EHFT app once their sample was processed, which instructed them to refer to the study instructions for completing their reference sample and contact their health care provider if they were concerned about their symptoms.

Participants were also asked to obtain a second midturbinate swab using the swab included in their influenza kit, following written and photographic instructions on both the quick start guide and the REDCap survey (Multimedia Appendix 1). They were instructed to insert the swab halfway (approximately 1 inch) into either nostril, press against the side, and rotate 5 times. They were then instructed to place the swab into the collection tube, repackage all components to meet US regulations for shipping biological substances (UN3373 Category B) [23], and return via the US Postal Service to the University of Washington research laboratory.

Participants received a follow-up survey 7 days after enrollment, which included questions about their illness duration and severity, recent travel, and feedback for the research team (Multimedia Appendix 1).

### Reference Testing

Returned kits received in the laboratory were examined, and any evidence of damage to the sample or packaging was documented. Samples were split into 2 aliquots of 1 mL. One aliquot was frozen at −80 ℃, and the other was stored at 4 ℃ until extraction. All samples were run in duplicate. Approximately 200 µL of VTM were extracted using Magna Pure 96 small-volume total nucleic acids extraction kit (product #06543588001; Roche). Purified total nucleic acids were tested against a panel of respiratory pathogens using the TaqMan OpenArray platform (Thermofisher) for qRT-PCR. The OpenArray panel included probe sequences for influenza A H3N2 and influenza A H1N1 and pan influenza A; influenza B; influenza C; respiratory syncytial virus (RSV) A and B; human coronavirus 229E, NL63, OC43, and HKU1; adenovirus; human rhinovirus; human metapneumovirus; human parechovirus; enterovirus A, B, C, D, D68, and G; human bocavirus; and *Streptococcus pneumoniae*, *Mycoplasma pneumoniae*, and *Chlamydia pneumoniae*. The OpenArray panel also included probes for the human gene ribonuclease P (RNase P) as an indicator of sample quality. All quantitative data were captured as relative cycle threshold (Crt) values, which is approximately 10 cycles less than the equivalent quantitative polymerase chain reaction cycle threshold.

Laboratory personnel did not have access to EHFT results or clinical information when interpreting the reference assay. A reference test was considered positive for a pathogen if qRT-PCR generated a fluorescent signal for the channel-specific pathogen within 40 polymerase chain reaction (PCR) cycles*.* The EHFT or laboratory results were not visible to the participants.

In May 2020, following the completion of data collection, participants were asked if they wanted to opt in to receive the results of their reference swab. If participants opted for results, the second aliquot of their sample was thawed and tested using the Clinical Laboratory Improvement Amendments–waived GeneXpert Xpress (Cepheid) with *Xpert Xpress Flu/RSV* cartridges, following the manufacturer’s instructions. Cepheid results distinguished influenza A, influenza B, and RSV. Participants were notified if influenza or RSV were detected. In addition, participants who consented to the study between March 4 and March 9 were notified in real time if SARS-CoV-2 was detected in their reference sample.

### Data Analysis

A participant flow diagram was created demonstrating each major step in the study and participant dropout. Summary statistics were calculated for participant demographics, risk factors, ARI symptoms, symptom severity, and other detected pathogens. Pearson chi-square test with Yate continuity correction was calculated for risk factors, symptom presence, and symptom severity between participants who were reference test positive (PCR positive) and negative (PCR negative). *P* values <.05 were considered statistically significant. We calculated symptom onset as the difference between the self-reported symptom onset date and the exact time at which the EHFT was completed. Participants were instructed to collect the reference swabs immediately after taking the EHFT.

We calculated the sensitivity, specificity, and positive and negative likelihood ratios (with 95% CI) for the overall performance of the index test (EHFT) compared with the reference test (OpenArray qRT-PCR) and independently for influenza A and B. In addition, we analyzed data by subgroups that had previously been shown to affect viral load [24-26], namely symptom onset before testing and illness severity measured as the total number of symptoms (1-9 symptoms) and disruption of daily life caused by their illness (1-5 scale, 1 being not at all and 5 being very much). For each subgroup, we calculated the sensitivity, specificity, and positive and negative likelihood ratios with a 95% CI. We performed pairwise comparisons of the mean level of impact on activities between subgroups using 1-way analysis of variance and Tukey honestly significant difference test. Where appropriate, Pearson correlations were calculated.

The average influenza Crt value was used as a proxy for relative viral load; lower Crt values correspond to higher viral loads and, thus, fewer cycles to generate a sufficient OpenArray signal [27]. Each additional Crt cycle is equivalent to a roughly 2-fold reduction in the genomic copies of viral RNA. Means and SDs were calculated for the following subgroups: symptom onset, influenza subtype, child and adult (5-17 years vs ≥18 years), and true positive (TP) versus false negative (FN) subgroups. Pairwise comparisons of mean influenza Crt for the subgroups were performed using the Student 2-tailed *t* test. Multiple linear regression models were fitted separately for average Crt values as a function of symptom onset, adjusted for age and number of symptoms and their level of impact on daily activities, both adjusted for age and symptom onset.

The RNase P Crt value was used as an indicator of reference sample quality; a lower Crt corresponds to more endogenous human DNA in the sample, indicating a greater likelihood of sufficient material collected on the swab [25,28,29]. Median RNase P Crt values were compared between age groups and between TP, false positive (FP), FN, and true negative test result subgroups using a Kruskal-Wallis test on ranks and Dunn multiple comparisons post hoc test with a Holm–Bonferroni correction. Median RNase P Crt values were compared between PCR-positive and PCR-negative groups, influenza A–positive and influenza B–positive groups, and between child (aged ≤18 years) and adult (aged >18 years) groups using the Mann-Whitney *U* test.

Participants with missing or indeterminate EHFT or reference samples were removed from the analysis. The analysis was conducted using R (version 1.3.1056; R Foundation for Statistical Computing) [30].

## Results

### Participant Recruitment and Retention

**Figure 1:**
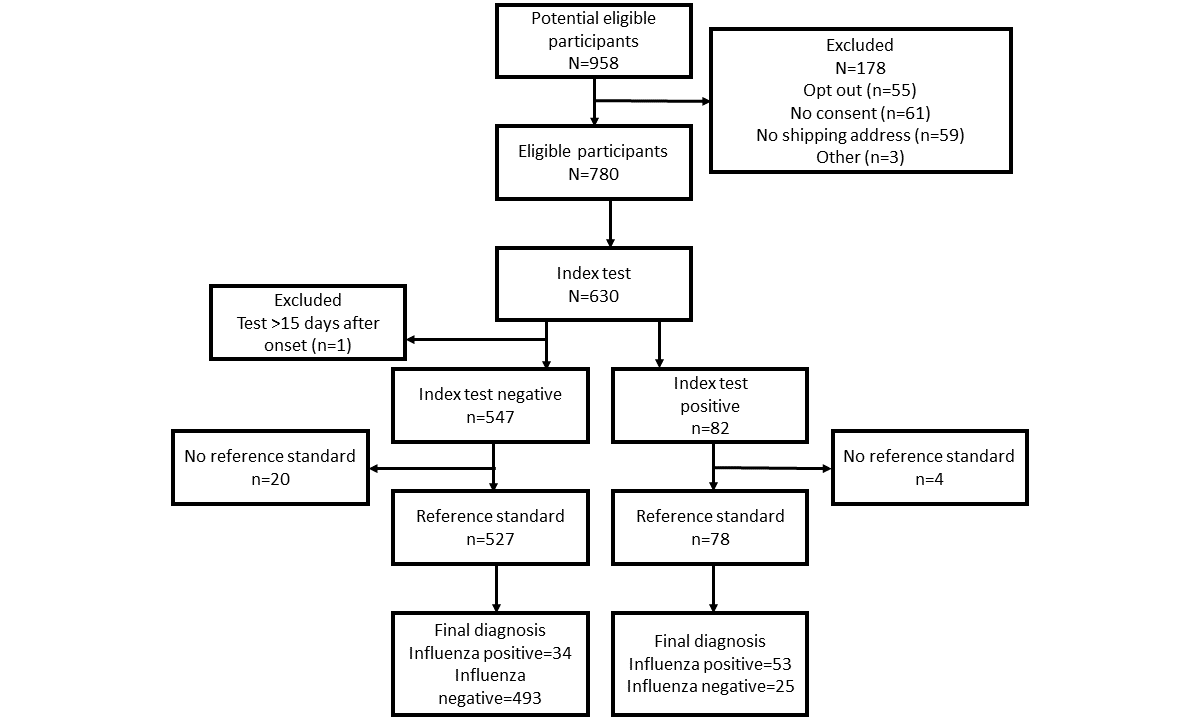
Recruitment flow for the Home Flu Test participants in Seattle, Washington.

A total of 958 participants met the inclusion criteria (Figure 1), of whom 780 (81.4%) completed the consent form, provided a viable shipping address, and were sent an influenza kit. Of these 780 participants who received their kit, 630 (80.8%) completed the index test. One of the individuals who completed the index test 34 days after symptom onset was excluded. Of those 630 participants who completed the index test, 605 (96%) returned their reference samples to the laboratory and were included in our analysis. The final study sample included in this analysis was the 605 participants who completed both the index and reference tests.

Most participants were recruited through advertisements on Facebook, Instagram, or Twitter (184/605, 30.4%); friend or family referrals (181/605, 29.9%); and other web-based media (138/605, 22.8%). Google advertisements and health care provider referrals recruited more influenza-positive participants than other recruitment methods.

### Participant Demographics

Most participants were aged 25-44 years, female, White, and had completed a bachelor’s degree or more (Table 1). Most had private health insurance, had received the 2019 influenza vaccination, and were nonsmokers. Age (*P*=.02) and education (*P*=.02) were significantly associated with a positive influenza PCR test; however, receipt of the 2019-2020 influenza vaccine was not associated with a positive influenza PCR test. The most frequent symptoms reported were fatigue (438/605, 72.4%), sore throat (414/605, 68.4%), cough (355/605, 58.7%), headache (342/605, 56.5%), and fever (292/605, 48.3%; Table 2). Fever, cough, chills or shivering, sweats, nausea or vomiting, myalgia (all *P* values <.001), fatigue (*P*=.007), runny nose (*P*=.04), and difficulty breathing (*P*=.05) were all significantly associated with a positive PCR test. Participants with positive influenza PCR were also more likely to report moderate or severe levels of fever (*P*<.001), cough (*P*<.001), fatigue (*P*<.001), myalgia (*P*<.001), and sore throat (*P*=.04) compared with those with a negative PCR test. The TaqMan OpenArray identified 30.7% (186/605) of participants who tested positive for ≥1 respiratory pathogen other than influenza, some of which were coinfections with influenza A or B (Multimedia Appendix 2). Other than influenza, the most common respiratory pathogens identified were rhinovirus (77/186, 41.2%) and seasonal human coronavirus (50/186, 26.9%).

Almost all (604/605, 99.8%) participants completed the EHFT within 15 days of symptoms onset (range 0.6-14.4 days; Multimedia Appendix 2), with an average time from symptom onset to EHFT testing of 2.9 (SD 1.5) days. Of the total 605 participants, 344 (56.9%) took the EHFT within 72 hours (3 days) after symptom onset, 249 (41.2%) between 4 and 7 days, and 12 (1.9%) between 8 and 15 days after symptom onset. The longest median time interval segment between symptom onset and EHFT testing was between symptom onset and study enrollment (median 48 hours) compared with time from enrollment to kit shipping (median 1.25 hours) and from kit shipping to testing (median 5.33 hours).

**Table 1:** Characteristics of participants with and without influenza detected on reference test (N=605).

Demographics		Overall, n (%)	Influenza PCR^a^ positive (n=87), n (%)	Influenza PCR negative (n=518), n (%)	*P* value
**Age (years)**					
	5-12	29 (4.8)	10 (13.8)	19 (3.7)	.02
	13-17	4 (0.7)	1 (1.1)	3 (0.6)	.02
	18-24	54 (8.9)	6 (6.9)	48 (9.3)	.02
	25-34	222 (36.8)	23 (26.4)	199 (38.4)	.02
	35-44	159 (26.2)	26 (29.9)	133 (25.7)	.02
	45-64	112 (18.5)	18 (20.7)	94 (18.1)	.02
	≥65	12 (2)	1 (1.1)	11 (2.1)	.02
**Gender**					
	Male	217 (36)	35 (40.2)	182 (35.1)	.07
	Female	360 (59.4)	47 (54)	313 (60.4)	.07
	Other	4 (0.7)	2 (2.3)	2 (0.4)	.07
**Education**					
	Less than high school	3 (0.5)	2 (2.3)	1 (0.2)	.02
	Graduated high school or GED^b^	15 (2.5)	0 (0)	15 (2.9)	.02
	Some college	55 (9.2)	5 (5.7)	50 (9.7)	.02
	Bachelor’s degree	234 (38.6)	37 (42.5)	197 (38)	.02
	Advanced degree	236 (38.9)	28 (32.2)	208 (40.2)	.02
**Race^c^**					
	White	430 (71)	69 (79.3)	361 (69.7)	.11
	Black	9 (1.5)	3 (3.4)	6 (1.2)	.25
	Asian	137 (22.8)	10 (11.5)	127 (24.5)	.01
	AI^d^, AN^e^, NH^f^, or PI^g^	10 (1.7)	0 (0)	10 (1.9)	N/A^h^
	Other	21 (3.5)	3 (3.4)	18 (3.5)	.99
Hispanic		31 (5.1)	2 (2.3)	29 (5.6)	.31
**Health insurance^c^**					
	Public	42 (6.9)	4 (4.6)	38 (7.3)	.48
	Private	519 (85.8)	76 (87.4)	443 (85.5)	.91
	Other	7 (1.2)	1 (1.1)	6 (1.2)	.99
	No insurance	11 (1.8)	3 (3.4)	8 (1.5)	.43
Taken 2019-2020 influenza vaccination		424 (70)	62 (71.3)	362 (69.9)	.78
Not a current smoker		516 (85.3)	71 (81.6)	445 (85.9)	.44

^a^PCR: polymerase chain reaction.

^b^GED: General Education Development.

^c^Total values may exceed 100% as participants could select multiple responses.

^d^AI: American Indian.

^e^AN: Alaskan Native.

^f^NH: Native Hawaiian.

^g^PI: Pacific Islander.

^h^N/A: not applicable.

**Table 2:** Presence and severity of symptoms reported by participants with and without influenza detected on reference test (N=605).

Symptoms		Overall, n (%)	Influenza PCR^a^ positive (n=87), n (%)	Influenza PCR negative (n=518), n (%)	*P* value
**Feeling feverish^b^**		292 (48.3)	76 (87.4)	216 (41.7)	<.001
	Mild	119 (19.7)	14 (16.1)	105 (20.3)	<.001
	Moderate	122 (20.2)	34 (39.1)	88 (17)	<.001
	Severe	51 (8.4)	28 (32.2)	23 (4.4)	<.001
Headache		342 (56.5)	63 (72.4)	279 (53.9)	.002
**Cough^b^**		355 (58.7)	72 (82.8)	283 (54.6)	<.001
	Mild	168 (27.8)	17 (19.5)	151 (29.2)	<.001
	Moderate	150 (24.8)	37 (42.5)	113 (21.8)	<.001
	Severe	36 (6)	18 (20.7)	18 (3.5)	<.001
Chills or shivering		227 (37.5)	68 (78.2)	159 (30.7)	<.001
Sweats		142 (23.5)	43 (49.4)	99 (19.1)	<.001
**Sore, itchy, or scratchy throat^b^**		414 (68.4)	57 (65.5)	357 (68.9)	.53
	Mild	174 (28.8)	27 (31)	147 (28.4)	.04
	Moderate	189 (31.2)	18 (20.7)	171 (33)	.04
	Severe	49 (8.1)	11 (12.6)	38 (7.3)	.04
Nausea or vomiting		72 (11.9)	21 (24.1)	51 (9.8)	<.001
Running or stuffy nose		358 (59.2)	61 (70.1)	297 (57.3)	.04
**Feeling more tired than usual^b^**		438 (72.4)	74 (85.1)	364 (70.3)	.007
	Mild	105 (17.4)	11 (12.6)	94 (18.1)	<.001
	Moderate	217 (35.9)	27 (31)	190 (36.7)	<.001
	Severe	116 (19.2)	36 (41.4)	80 (15.4)	<.001
**Muscle or body aches^b^**		300 (49.6)	65 (74.7)	235 (45.4)	<.001
	Mild	99 (16.4)	4 (4.6)	95 (18.3)	<.001
	Moderate	146 (24.1)	37 (42.5)	109 (21)	<.001
	Severe	55 (9.1)	24 (27.6)	31 (6)	<.001
Increased trouble with breathing		134 (22.1)	27 (31)	107 (20.7)	.047
Diarrhea^c^		69 (11.4)	6 (6.9)	63 (12.2)	.19
Rash^c^		9 (1.5)	0 (0)	9 (1.7)	.44
Ear pain or discharge^c^		54 (8.9)	11 (12.6)	43 (8.3)	.31

^a^PCR: polymerase chain reaction.

^b^Included survey questions on severity for a subset of symptoms presented in this table.

^c^Symptoms reported for children (aged <18 years) only.

### Accuracy of RDT

Of the total 605 participants, 87 (14.4%) participants tested positive for influenza using the reference test (70/87, 80% for influenza A and 17/87, 20% for influenza B). The overall sensitivity and specificity of the EHFT compared with the reference test were 61% (95% CI 50-71) and 95% (95% CI 93-97), respectively (see Table 3 for accuracy values). The sensitivity and specificity of the EHFT for influenza A were 60% (95% CI 48-72) and 99% (95% CI 98-100), respectively, and those for influenza B were 65% (95% CI 38-86) and 96% (95% CI 94-98), respectively. Influenza B yielded a higher rate of FP tests, which resulted in a positive predictive value (PPV) of 33% (95% CI 18-52).

Subgroup analysis of EHFT accuracy based on the time from symptom onset to conducting the EHFT (Table 3) showed that the sensitivity and specificity of influenza A detection at ≤72 hours of symptom onset were 62.5% (95% CI 48-77) and 100% (95% CI 98-100), respectively, whereas at >72 hours they were 57% (95% CI 37-75) and 99% (95% CI 97-100), respectively. The sensitivity and specificity of influenza B at ≤72 hours were 64% (95% CI 31-89) and 95% (95% CI 92-97), respectively, whereas, at >72 hours, they were 67% (95% CI 22-96) and 98% (95% CI 95-99), respectively.

There were no associations between illness severity and EHFT sensitivity (Multimedia Appendix 3). Neither the number of symptoms nor disruption to daily activities were significantly associated with EHFT sensitivity, nor was there a meaningful association between either of these measures and mean influenza Crt. However, measures of illness severity were correlated with each other; individuals who reported more disruption to daily activities also reported a greater number of symptoms (*r*=0.54; *P*<.001), and individuals who were PCR positive reported significantly higher scores for both these measures compared with individuals who were PCR negative (*P*<.001; Multimedia Appendix 3).

Of the 25 FP results, 22 (88%) occurred when the EHFT indicated the presence of influenza B, and all occurred within 96 hours of symptom onset (Multimedia Appendix 3). Other respiratory pathogens, namely RSV, human metapneumovirus*,* human coronavirus*,* and *Streptococcus pneumoniae*, were detected in 32% (7/22) of the influenza B FP samples.

**Table 3:** Accuracy of the Ellume home influenza test compared with laboratory reference polymerase chain reaction values (N=605).

Accuracy			True positive, n (%)		False positive, n (%)		False negative, n (%)		True negative, n (%)		Sensitivity (95% CI)		Specificity (95% CI)		PPV^a^ (95% CI)		NPV^b^ (95% CI)
**Overall**																	
	All	53 (8.8)		25 (4.1)		34 (5.6)		493 (81.5)		61 (50-71)		95 (93-97)		68 (56-78)		94 (91-95)	
	≤72 hours (n=344)	32 (9.3)		17 (4.9)		19 (5.5)		276 (80.2)		63 (48-76)		94 (91-97)		65 (50-78)		94 (90-96)	
	>72 hours (n=261)	21 (8)		8 (3.1)		15 (5.7)		217 (83.1)		58 (41-74)		96 (93-98)		72 (53-87)		94 (90-96)	
**Influenza A**																	
	All	42 (6.9)		3 (0.5)		28 (4.6)		532 (87.9)		60 (48-72)		99 (98-100)		93 (82-99)		95 (93-97)	
	≤72 hours (n=344)	25 (7.3)		1 (0.3)		15 (4.4)		303 (88.1)		63 (46-77)		100 (98-100)		96 (80-100)		95 (92-97)	
	>72 hours (n=261)	17 (6.5)		2 (0.8)		13 (5)		229 (87.7)		57 (37-75)		99 (97-100)		89 (67-99)		95 (91-97)	
**Influenza B**																	
	All	11 (1.8)		22 (3.6)		6 (1)		566 (93.6)		65 (38-86)		96 (94-98)		33 (18-52)		99 (98-100)	
	≤72 hours (n=344)	7 (2)		16 (4.7)		4 (1.2)		317 (92.2)		64 (31-89)		95 (92-97)		30 (13-53)		99 (97-100)	
	>72 hours (n=261)	4 (1.5)		6 (2.3)		2 (0.8)		249 (95.4)		67 (22-96)		98 (95-99)		40 (12-74)		99 (97-100)	

^a^PPV: positive predictive value.

^b^NPV: negative predictive value.

### Influenza Crt

The average influenza Crt value for all influenza PCR-positive samples was 18.82 and was significantly lower for samples collected ≤72 hours after symptom onset (17.9, SD 4.4), than those collected at >72 hours (20.14, SD 4.2; *P*=.02; Table 4). The mean influenza Crt (16.8, SD 4.0) for individuals who were TP was significantly lower than the mean Crt value (22.0, SD 3.0) for individuals who were FN (*P*<.001). The mean influenza Crt values were also significantly lower for influenza B (16.7, SD 4.4) than for influenza A (19.3, SD 4.3; *P*=.04). Multiple regression estimated that the average Crt increased by 1.34 (95% CI 39-2.29) Crt for each additional day after symptom onset (*P*=.006), and Crt values decreased with a greater number of symptoms (*P*=.03; Multimedia Appendix 4).

**Table 4:** Influenza relative cycle threshold (Crt) of various subgroups (N=87).

Group			Influenza-positive participants, n (%)		Influenza Crt raw, mean (SD)		*P* value
**Influenza subtype**							
	Influenza A	70 (80)		19.34 (4.3)		.04	
	Influenza B	17 (20)		16.71 (4.4)		.04	
**72-hour testing cutoff**							
	≤72 hours	N/A^a^		17.9 (4.4)		.02	
	>72 hours	N/A		20.14 (4.2)		.02	
**Age^b^ (years)**							
	5-17	11 (13)		16.6 (4.5)		.10	
	≥18	73 (84)		19.1 (4.4)		.10	
**True positives and false negatives**							
	True positives	53 (61)		16.8 (4.0)		<.001	
	False negatives	34 (39)		22.0 (3.0)		<.001	

^a^N/A: not available.

^b^A total of 3 participants who were influenza positive were missing age data.

### User Experiences With Study Procedures and Specimen Quality

Overall, participants stated they were *somewhat confident* (216/560, 38.5%) or *very confident* (337/560, 60.1%) that they completed the reference swab correctly and experienced only mild discomfort (431/560, 76.8%; Multimedia Appendix 5). This was similar for the EHFT, for which participants stated they were *somewhat confident* (188/567, 33.5%) or *very confident* (371/567, 66.1%) and experienced only mild discomfort (427/567, 76.1%). Only 0.3% (2/567) of participants reported errors with the Bluetooth component of the EHFT device and were sent new devices. No other issues with the device were reported to the study team.

Reference sample RNase P Crt values ranged from 10.89 cycles to 33.3 cycles (median 21.1, SD 4.3, IQR 16.7-24.1). RNase P Crt did not vary significantly between influenza-positive samples (median 22.7, SD 4.32) and influenza-negative samples (median 20.7, SD 4.32; *P*=.05) or based on age or influenza subtype (Multimedia Appendix 5). All samples had Rnase P Crt values well below the 40-cycle cutoff value, and only 4 were >30 (31.5, 32.4, 32.5, and 33.3). Median values for all subgroups assessed were well within the recommended quality range of 28 Crt [31]. No samples were excluded on the basis of their RNase P Crt values.

In 7.8% (46/587) of the returned kits, ≥1 error was noted, indicating that these participants did not correctly follow ≥1 provided instruction. Of these 46 kits, 23 (50%) were returned with a packaging error (either missing the outer box or specimen bag sealed incorrectly), and 27 (59%) were returned with incorrect labeling on the VTM tube (Multimedia Appendix 5).

## Discussion

### Principal Findings

This study demonstrated the feasibility of implementing an unsupervised at-home diagnostic test. The vast majority of participants were able to complete the multiple procedures required to evaluate a home influenza test without direct supervision, including surveys, 2 midturbinate swabs, app-guided directions to complete an influenza RDT, and returning reference samples by mail to a central laboratory. The influenza positivity within our study sample was 14.4% (87/605), with 80% (70/87) of influenza A cases, which is consistent with both the prevalence and relative proportion of influenza strains reported in the local area during the study period [32]. The EHFT had moderate sensitivity (61%) and high specificity (95%) compared with laboratory PCR on self-collected swabs. Specificity was slightly higher (99%) for influenza A than for influenza B (96%), whereas sensitivity was slightly higher (65%) for influenza B than for influenza A (60%), although the CIs were wide and overlapping. The small proportion of participants who were PCR positive for influenza B and the high rate of influenza B FPs resulted in a much lower PPV for influenza B (33%) when analyzed independently from influenza A (68%).

TP EHFT results had significantly lower influenza Crt values (corresponding to higher viral load) than FN EHFT results, suggesting that lower viral load may have affected the sensitivity of EHFT. To further investigate this relationship, we assessed EHFT accuracy across 2 additional variables known to affect viral load, namely, symptom onset and illness severity [24-26,33,34]. EHFT sensitivity was related to symptom onset, with a moderate improvement in sensitivity (6%) when the test was conducted within 72 hours of symptom onset. Furthermore, we noted a linear relationship between symptom onset and Crt value, where each additional day between symptom onset and testing corresponded to an average of 1.3 additional cycles (ie, more than a 2-fold decrease) in the estimated quantity of the virus. In contrast, neither did illness severity appear to influence EHFT sensitivity, with no relationship observed between test accuracy and number of symptoms, nor did it have a greater impact on daily activities. Nevertheless, these 2 measures of illness severity were correlated with each other, which is consistent with the expectation that individuals who report more symptoms face greater disruption to their daily activities. Notably, both measures of influenza severity were significantly higher in individuals who were PCR positive than in individuals who were PCR negative, despite not predicting viral load in this study.

We did not find any evidence that the quality of self-sampling affected EHFT accuracy or that particular demographic groups were more capable of collecting a self-swab than others. In contrast, we found that negative reference swabs had a lower RNase P Crt value than the positive samples. If sample quality affected the reference swab, we would expect the opposite—negative reference swabs to be of poorer quality and thus higher RNase P Crt values. The median RNase *P* values for the test accuracy subgroups were well within the acceptable range.

### Comparison With Prior Studies

The accuracy of the EHFT we reported was comparable with that of other influenza RDTs. A 2017 meta-analysis of 134 studies of influenza RDTs showed pooled estimates of sensitivity (61%, 95% CI 53.3-68.3) and specificity (98.9%, 95% CI 98.4-99.3) [14] that are comparable with those reported in this study. These results are consistent with those of 2 other meta-analyses [10,14]. The similarity in test accuracy is even more notable considering that published studies on influenza RDT accuracy were conducted in health care settings, with sampling and RDTs performed by health care workers and researchers rather than patients themselves.

The time from symptom onset to testing, or symptom onset, has a critical impact on viral load and influenza RDT accuracy [13,35]. One of the disadvantages of mailed testing kits is the delay in testing following symptom onset because of the time needed to distribute swabbing materials [16,36]. Elliot et al [36] reported an average of 4 days between symptom onset and self-swabbing compared with 2 days for clinician-collected samples. Similarly, we found that influenza Crt decreased with a longer symptom onset [36]. Studies that have lower mean times from onset to testing tend to report higher sensitivity values [13]. In contrast, 1 study found that testing too early can lead to increased FNs, primarily if the RDT is used within 12 hours of symptom onset [15], suggesting that there might be a *sweet spot* for RDT testing for influenza that must be balanced with other factors that affect test sensitivity. In addition, we did not find a relationship between viral shedding and illness severity [24-26,33,34]. This may be as the measures of illness severity we used lacked validity in our setting or population, and the range of illness severities in our population was too narrow. A more robust understanding of viral load dynamics, especially in less severely ill populations, will help delineate the conditions under which an at-home RDT for influenza is most appropriate.

The quality of self-collected swabs did not appear to affect the EHFT accuracy. Participants reported high confidence in completing both the EHFT swab and reference test swab. Moreover, reference swabs had RNase P Crt values (when corrected to an equivalent cycle threshold value) that tended to be high but within the range of those reported in other studies of both clinician-collected and self-collected midturbinate swabs [28,37]. The EHFT swab instructions asked the participants to swab both nostrils. A possible explanation for the low RNase P Crt values on the reference swab, which was completed after the EHFT swab, is that there was less human cellular debris available for collection. This may also have affected the influenza Crt values. Although there was variability in RNase P Crt between individuals, variability was not observed between TP, FN, FP, and true negative groups, suggesting that sample collection did not affect EHFT accuracy.

Our findings of inferior accuracy of the EHFT for influenza B (including FP results) are consistent with other literature [38-40] and may have been because of several factors. The prevalence of influenza B in the study catchment area was low (3%-4%) during the study period [32]; low disease prevalence is known to affect predictive values [41,42]. Other studies of influenza RDTs have also noted higher rates of FPs for influenza B than influenza A [38-40], suggesting nonspecific reactivity with antibodies used for influenza B detection.

### Strengths and Limitations

This is one of the first studies to report the accuracy of an influenza RDT used by unsupervised participants and the first to do so for an RDT designed specifically for home use. Our study design included remote web-based recruitment, shipping of influenza kits complete with RDT and reference sample collection materials, and completion of all stages of the study by the user without direct supervision from the study staff. The high response and completion rate of study procedures (605/780, 77.6%) were matched by high self-reported confidence for both the EHFT and reference sample procedures. There was a 22.4% (175/780) dropout of participants who were sent a kit but did not participate; it is unclear whether this introduced additional bias but is consistent with other mail-based testing studies [43]. Although occasional errors in the required shipping procedures occurred, the vast majority of samples were returned to the research laboratory in the appropriate condition. Participants likely had milder symptoms than those attending clinical settings, and although this may have affected RDT sensitivity with lower viral shedding, they represented the population in which this RDT would be used. Our findings support this type of study design for the assessment of self-tests for influenza and other respiratory viruses such as SARS-CoV-2.

This study had several limitations. First, participants were more highly educated, were English speakers with private insurance and access, and had the ability to use a Bluetooth mobile app device. Future studies may address this limitation through varied recruitment approaches outside of the social media advertisements used in this study; however, this type of study design depends on internet- and app-based data collection, which inherently limits the study population. Second, many individuals (261/605, 43.1%) did not conduct the EHFT within 72 hours of illness onset, likely because of the time elapsed from recruitment, shipment of kits, and participants’ availability to complete the EHFT on receipt. Future studies of at-home tests should explore solutions to identify symptomatic individuals earlier in their illness and expediently provide tests. For example, cohort studies have used regular, self-reported symptom surveys via SMS text messaging or email to identify influenza early and prompt testing [44,45] or through prepositioning of influenza kits or other at-home testing devices. Third, although participants reported confidence in self-collection of swabs, confirmed by markers of human DNA in these samples, there remains some uncertainty regarding the validity of this type of reference sample. Finally, we recruited less than one-third of the desired sample size (and only a small number of influenza B infections) because of delays in study initiation and premature closure as a result of the COVID-19 pandemic in the local area. A larger sample size would have provided tighter CIs around the estimates of test accuracy. In addition, it is possible that the circulation of SARS-CoV-2 in the community from which we recruited affected the accuracy of the EHFT. The enrollment criteria were intended to be specific to ARI and thus may have incidentally recruited individuals with COVID-19 who presented with symptoms similar to influenza*.* Unfortunately, because of privacy protocols implemented by our laboratory in conjunction with the county public health office, we were not able to analyze the reference samples for SARS-CoV-2 and were unable to determine the impact it may have had on the EHFT accuracy. On the basis of the seroprevalence of SARS-CoV-2 around this time [46], it seems unlikely that there were enough COVID-19 cases circulating in the Seattle area to cause a major reduction in test performance, although it may have had a small impact on PPV for ARI.

### Implications for Clinicians, Researchers, and Policy Makers

RDTs designed for home use have the potential to be purchased over the counter or prescribed by health care providers and coupled, if necessary, with in-person or telemedicine consultations to guide care [47]. The accuracy of the influenza EHFT reported here is similar to that of many RDTs used in clinical settings, which supports its use in similar populations, provided suitable precautions are in place, particularly to mitigate the risk of FN results. These could include using clinical prediction rules to assist potential RDT users in quantifying their pretest probability of influenza. Our findings support the use of the EHFT among individuals within 72 hours of symptom onset and suggest the need for further research to understand other indicators of viral load that could be used to select individuals for whom this type of RDT should or should not be recommended.

Our study design provides a model for comparative accuracy studies of RDTs for influenza and other respiratory pathogens, including SARS-CoV-2, in home settings. We recommend that future study designs prioritize minimizing the time from symptom onset through study enrollment to conduct the index test, particularly for infections such as influenza, where viral shedding declines rapidly after symptom onset. Strategies could include prepositioning test kits and encouraging early completion of RDT with the onset of illness. Given the potentially important relationship between influenza severity and viral load (and hence self-test sensitivity), we also encourage the use of more accurate ways of measuring illness severity from self-reported surveys. Finally, rather than blinding participants to RDT results, revealing self-test results would facilitate recruitment and allow exploration of the impacts of positive and negative self-test results on participants’ health-seeking and other behaviors.

### Conclusions

Using an entirely community-based, remote recruitment study design, our findings showed that the EHFT had comparable accuracy to many influenza RDTs used in clinical settings. However, the sensitivity of the EHFT was only moderate and was higher when the test was used within 72 hours of symptom onset when virus shedding was likely the highest. Our findings support a new form of trial design, in which recruitment and self-sampling for reference testing can be performed successfully by lay users in the communities and populations in which these tests will be implemented. Such study designs could be used to assess the accuracy of tests for other viral respiratory tract pathogens, such as SARS-CoV-2 and RSV. Home tests have the potential to expand access to testing for infectious diseases, with potential benefits for individuals and the health care system.

## Appendix

Multimedia Appendix 1 Additional details on participant data collection.

Multimedia Appendix 2 Additional participant demographics.

Multimedia Appendix 3 Additional details on rapid diagnostic test accuracy.

Multimedia Appendix 4 Regression adjusted mean influenza relative cycle threshold.

Multimedia Appendix 5 User experience with study procedures and specimen quality.

## Abbreviations

ARI: acute respiratory illness
Crt: relative cycle threshold
EHFT: Ellume Home Flu Test
FN: false negative
FP: false positive
PCR: polymerase chain reaction
POC: point-of-care
PPV: positive predictive value
RDT: rapid diagnostic test
REDCap: Research Electronic Data Capture
RNase P: ribonuclease P
qRT-PCR: quantitative reverse-transcription polymerase chain reaction
RSV: respiratory syncytial virus
SFS: Seattle Flu Study
STARD: Standards for Reporting of Diagnostic Accuracy Studies
TP: true positive
VTM: viral transport medium
