# Supplementary material for: Diagnostic Accuracy of an At-Home, Rapid Self-test for Influenza: Prospective Comparative Accuracy Study
Source: JMIR Public Health Surveill. 2022 Feb 22;8(2):e28268. doi: 10.2196/28268 (PMC8905479; doi:10.2196/28268)

# Multimedia Appendix 1

## Additional details on participant data collection

### Illness Questionnaire and Follow Up Survey

1. How long ago did you first notice your current illness? Select the date on the calendar.
2. Around when did your symptoms start?
   1. half a day ago
   2. half a day - 1 day ago
   3. 1-1.5 days ago
   4. 1.5-2 days ago
   5. 3 days ago
   6. 4 days ago
   7. 5 days ago
3. How long did it take you to go from feeling not sick at all to feeling the sickest you have felt?
   1. half a day ago
   2. half a day - 1 day ago
   3. 1-1.5 days ago
   4. 1.5-2 days ago
   5. 3 days ago
   6. 4 days ago
   7. 5 days ago
4. Which new or worsening symptoms have you experienced in the last 7 days? Select all that apply.
   1. Feeling feverish
   2. Headache
   3. Cough
   4. Chills or shivering
   5. Sweats
   6. Sore throat or itchy/scratchy throat
   7. Nausea or vomiting
   8. Runny or stuffy nose
   9. Feeling more tired than usual
   10. Muscle or body aches
   11. Increased trouble with breathing
   12. Diarrhea
   13. Rash
   14. Ear pain or ear discharge
   15. None of the above
5. How severe are your symptoms? (Select the level of discomfort you have felt at the worst point so far).
   1. Fever
      1. Mild
      2. Moderate
      3. Severe
   2. Cough
      1. Mild
      2. Moderate
      3. Severe
   3. Muscle or body aches
      1. Mild
      2. Moderate
      3. Severe
   4. Feeling more tired than usual
      1. Mild
      2. Moderate
      3. Severe
   5. Sore throat or itchy/scratchy throat
      1. Mild
      2. Moderate
      3. Severe
6. What is your sex
   1. Male
   2. Female
   3. Other (please specify)
   4. Prefer not to say
7. Are you Hispanic or Latino?
   1. Yes
   2. No
   3. Prefer not to say
8. How would you describe your race? (Select all that apply)
   1. American Indian or Alaska Native
   2. Asian
   3. Native Hawaiian or other Pacific Islander
   4. Black or African American
   5. White
   6. Other
   7. Prefer not to say
9. What is the highest level of education you have completed?
   1. Less than high school graduate
   2. Graduated high school/obtained GED
   3. Some college (including vocational training, associate's degree)
   4. Bachelor's degree
   5. Advanced degree
   6. Prefer not to say
10. Please choose the range that best represents your household income last year (before taxes). If you are still considered a "dependent" for tax purposes, choose the range that describes your parent/legal guardian's household income.
    1. Less than or equal to $25,000
    2. Between $25 and 50 thousand ($25,001 to $50,000)
    3. Between $50 and 75 thousand ($50,001 to $75,000)
    4. Between $75 and 100 thousand ($75,001 to $100,000)
    5. Between $100 and 125 thousand ($100,001 to $125,000)
    6. Between $125 and 150 thousand ($125,001 to $150,000)
    7. Over $150,000
    8. Don't know
    9. Prefer not to say
11. What type of health insurance do you have? Select all that apply.
    1. Private (provided by employer and/or purchased)
    2. Government
    3. Other
    4. None
    5. Prefer not to say
12. Are you currently pregnant?
    1. Yes
    2. No
    3. Prefer not to say
13. Are you affiliated with the University of Washington?
    1. Yes, I am an undergraduate student
    2. Yes, I am a graduate/professional student
    3. Yes, I am a faculty member
    4. Yes, I am a staff member/university employee
    5. No
14. What kind of residence do you live in?
    1. On-campus residence hall
    2. On-campus apartment
    3. Greek life housing
    4. Off-campus house
    5. Off-campus apartment
    6. Other
15. Where do you live?
    1. House/condo/townhouse
    2. Shelter
    3. Apartment
    4. Dormitory
    5. Assisted living facility
    6. Skilled nursing center
    7. No consistent primary residence
    8. Other
16. Including yourself, how many people share your kitchen or living space?
    1. I live by myself
    2. 2 people
    3. 3 people
    4. 4 people
    5. 5 people
    6. 6 or more people
17. What age groups of children are in your house? Select all that apply.
    1. No children
    2. Age 0-5 years
    3. Age 6-12 years
    4. Age 13-18 years
18. Do these children attend daycare or a child care center?
    1. Yes
    2. No
19. Approximately how many hours per week do these children attend daycare or childcare?
20. In the past 7 days, has someone you live with been diagnosed with the flu by a medical professional?
    1. Yes
    2. No
    3. Do not know
21. Have you sought clinical care for your current illness since it started?
    1. No
    2. Yes; Doctor's office or Urgent Care
    3. Yes; Pharmacy (drugstore)
    4. Yes; Hospital or Emergency Department
    5. Yes; Other
22. Are you receiving an antiviral prescribed by a medical professional for the treatment or prevention of flu?
    1. No
    2. Yes; Oseltamivir (Tamiflu)
    3. Yes; Zanamivir (Relenza)
    4. Yes; Peramivir (Rapivab)
    5. Yes; Baloxavir (Xofluza)
    6. Yes, but I don't know which medication
    7. Do not know
23. At my place of work, employees are encouraged to take time off or work from home if they are sick.
    1. Yes, and I would be paid for hours missed
    2. Yes, but I would not be paid for hours missed
    3. No
    4. I am not currently employed
24. How has your current illness affected your ability to do your regular activities (work, school, etc.)?
    1. Not at all
    2. A little bit
    3. Somewhat
    4. Quite a bit
    5. Very much
25. Other than work and/or school, which of the following daily activities have been impacted by your current illness? Select all that apply.
    1. Running errands
    2. Exercising
    3. Socializing
    4. Volunteering
    5. Ability to take care of myself and/or family
    6. None of the above/ my activities have not been impacted
26. Which of the following daily activities have been impacted by your current illness? Select all that apply.
    1. School
    2. Work
    3. Running errands
    4. Exercising
    5. Socializing
    6. Volunteering
    7. Ability to take care of myself and/or family
    8. None of the above/ my activities have not been impacted
27. Did any of the following occur because you were feeling sick? Select all that apply.
    1. I missed work
    2. I worked from home
    3. I worked fewer hours than usual
    4. None of the above
28. How many days were you not able to go to work?
29. Do you use any of the following products (either indoors or outdoors)?
    1. Tobacco products (e.g. cigarettes, cigars, pipes)
    2. Electronic cigarettes/vapor pens
    3. None of the above
    4. Prefer not to say
30. Does anyone in your shared living space use any of the following products (either indoors or outdoors)?
    1. Tobacco products (e.g. cigarettes, cigars, pipes)
    2. Electronic cigarettes/vapor pens
    3. None of the above
    4. Do not know
31. Have you received this season's influenza (flu) vaccine (since July 1, 2019)
    1. Yes
    2. No
    3. Do not know
32. What year did you get the flu shot or flu mist nasal spray this season (since July 1, 2019)
    1. 2019
    2. 2020
    3. Do not know
33. What month did you get the flu shot or flu mist nasal spray this season (since July 1, 2019)
    1. January
    2. February
    3. March
    4. April
    5. May
    6. June
    7. July
    8. August
    9. September
    10. October
    11. November
    12. December
    13. Do not know
34. How did you receive the flu vaccine this season (since July 1, 2019)
    1. Injection (flu shot)
    2. Nasal spray (flu mist)
35. Where did you receive the flu vaccine this season (since July 1, 2019)
    1. Medical clinic or hospital
    2. Pharmacy or drugstore
    3. Workplace
    4. School
    5. Other
36. What is the primary reason why you have not received the flu vaccine this season (since July 1, 2019)
    1. 1Do not have time to get vaccinated
    2. Not required for work or school
    3. Not recommended by a doctor or healthcare worker
    4. Not covered by health insurance
    5. Not offered at a convenient location
    6. I am not worried about getting sick with the flu
    7. Concerns about vaccine safety or effectiveness
    8. I plan to get the flu vaccine
    9. None of the above
37. Did you receive the flu vaccine last season? (July 1, 2018 - July 1, 2019)
    1. Yes
    2. No
38. Have you ever been told by a healthcare provider that you have one of the following medical conditions? Select all that apply.
    1. Asthma or reactive airway disease
    2. COPD/emphysema
    3. Chronic bronchitis
    4. Cancer
    5. Diabetes
    6. Heart disease (heart failure or heart attack)
    7. None of the above
    8. Do not know
    9. Prefer not to say
39. In the past 7 days, have you visited a country other than the US?
    1. No, I have not traveled outside the US
    2. Yes
40. Country visited (up to 5)
41. In the past 7 days have you traveled outside the state of Washington?
    1. No, I have not traveled outside of Washington
    2. Yes
42. State visited (up to 5)

### Follow Up (7 days after Illness Questionnaire)

1. Are you fully recovered from your illness?
   1. Yes
   2. No
   3. Do not know
2. How many days did your illness last?
3. Have you visited a country other than the US since [enrollment date]?
   1. No, I have not traveled outside the US
   2. Yes
4. Country visited (up to 5)
5. Have you traveled outside the state of Washington since [enrollment_date]?
   1. No, I have not traveled outside of Washington
   2. Yes
6. State visited (up to 5)
7. What was the purpose of your recent travel?
   1. Tourism
   2. Business
   3. Visiting family and/or friends
   4. I just moved here from another US state
   5. I just moved here from another country
   6. Other
8. When your recent illness was at its worst, how did it affect your ability to do your regular activities (work, school, etc.)
   1. Not at all
   2. A little bit
   3. Somewhat
   4. Quite a bit
   5. Very much
9. Were any of the following activities impacted because you were feeling sick? Select all that apply
   1. School
   2. Work
   3. Running errands
   4. Exercising
   5. Socializing
   6. Volunteering
   7. Ability to take care of myself and/or family
   8. None of the above/ my activities have not been impacted
10. Did your illness keep you from doing any of the following? Select all that apply.
    1. Attending class
    2. Going to work
    3. Studying
    4. Performing well on an exam or written assignment
    5. None of the Above/ My activities have not been impacted
11. How many days were you not able to go to school?
12. Did any of the following occur because you were feeling sick? Select all that apply.
    1. I missed work
    2. I worked from home
    3. I worked fewer hours than usual
    4. None of the above
13. How many days were you not able to go to work?
14. Did you seek clinical care (from a healthcare provider) for your illness since your symptoms were reported on [enrollment_date]?
    1. Yes - Doctor's office or Urgent Care
    2. Yes - Pharmacy (drugstore)
    3. Yes - Hospital or Emergency Department
    4. Yes - Other
    5. No
15. Did you receive an antiviral medication for your illness?
    1. No
    2. Yes; Oseltamivir (Tamiflu)
    3. Yes; Zanamivir (Relenza)
    4. Yes; Peramivir (Rapivab)
    5. Yes; Baloxavir (Xofluza)
    6. Yes, but I don't know which medication
    7. Do not know
16. Did you receive antibiotics for your illness?
    1. No
    2. Yes; Zithromycin (Z-pack or Zithromax)
    3. Yes; Amoxicillin (Moxatag)
    4. Yes; Amoxicillin/Clavulanate (Augmentin)
    5. Yes; Levofloxacin (Levaquin)
    6. Yes; Moxifloxacin (Avelox)
    7. Yes, but I don't remember which antibiotic
    8. Yes, but my antibiotic is not listed
    9. Do not know
17. Did you do any of the following because you enrolled in the Seattle Flu Study on [consent_date]? Select all that apply
    1. Washed hands/used hand sanitizer more frequently
    2. Avoided public transport
    3. Stayed home
    4. Wore a face mask
    5. Encouraged others to get the flu vaccine
    6. Avoided contact with others
    7. Other
18. Did you do any of the following in the past week because you were feeling sick? Select all that apply.
    1. More frequently washed hands with soap and water or used hand sanitizer more frequently than usual
    2. More frequently <b>covered my mouth and nose with a tissue when coughing or sneezing
    3. More frequently coughed or sneezed into my elbow or shoulder
    4. More frequently cleaned or disinfected my living space or workspace
    5. Avoided public transport or took it less often than I normally would
    6. Stayed home
    7. Wore a face mask in public to help protect others from getting sick
    8. Encouraged others to get the flu vaccine
    9. Avoided contact with others
    10. I did not change any of my behaviors because I was feeling sick
19. We would like your opinion on the Home Flu Test (kit and mobile app) that you used a week ago.
    1. I found the test easy to use
       1. Strongly Disagree
       2. Disagree
       3. Neither agree nor disagree
       4. Agree
       5. Strongly Agree
    2. I found the instructions on the app easy to use
       1. Strongly Disagree
       2. Disagree
       3. Neither agree nor disagree
       4. Agree
       5. Strongly Agree
    3. I feel confident using the test at home
       1. Strongly Disagree
       2. Disagree
       3. Neither agree nor disagree
       4. Agree
       5. Strongly Agree
20. Imagine there was a pandemic outbreak of influenza (flu) in the US and you were sick with cold- or flu-like symptoms. Please tick the box(es) that best match what you would do in the situations described below
    1. You obtained the Home Flu Test and tested positive for the flu
       1. I would minimize contact with others
       2. I would not go to work or school
       3. I would use the phone consultation service to obtain treatment and advice
       4. I would not change my behaviors
    2. You obtained the Home Flu Test and tested negative for flu
       1. I would minimize contact with others
       2. I would not go to work or school
       3. I would use the phone consultation service to obtain treatment and advice
       4. I would not change my behaviors
    3. You are sick with cold- or flu-like symptoms, but you did not do a Home Flu Test and did not receive a diagnosis
       1. I would minimize contact with others
       2. I would not go to work or school
       3. I would use the phone consultation service to obtain treatment and advice
       4. I would not change my behaviors
21. I believe it could save time to do a home-based test like Home Flu Test before visiting a healthcare provider.
    1. Strongly Disagree
    2. Disagree
    3. Neither agree nor disagree
    4. Agree
    5. Strongly Agree
22. I feel that the Home Flu Test could help me better manage my illness.
    1. Strongly Disagree
    2. Disagree
    3. Neither agree nor disagree
    4. Agree
    5. Strongly Agree
23. If the Home Flu Test becomes commercially available I would do the following (select all that apply):
    1. Share your results with a healthcare provider
    2. Share your results anonymously with a national flu tracking system
    3. Purchase a kit to have at home before you got sick
    4. Prefer to order a kit online when I have symptoms
    5. Prefer to purchase a kit at a pharmacy/drugstore when I have symptoms
    6. None of the above
24. Do you have any improvement suggestions for the Home Flu Test?
25. Is there anything else about your Seattle Flu Study experience that you'd like to share with us?

### Quick Start Guide


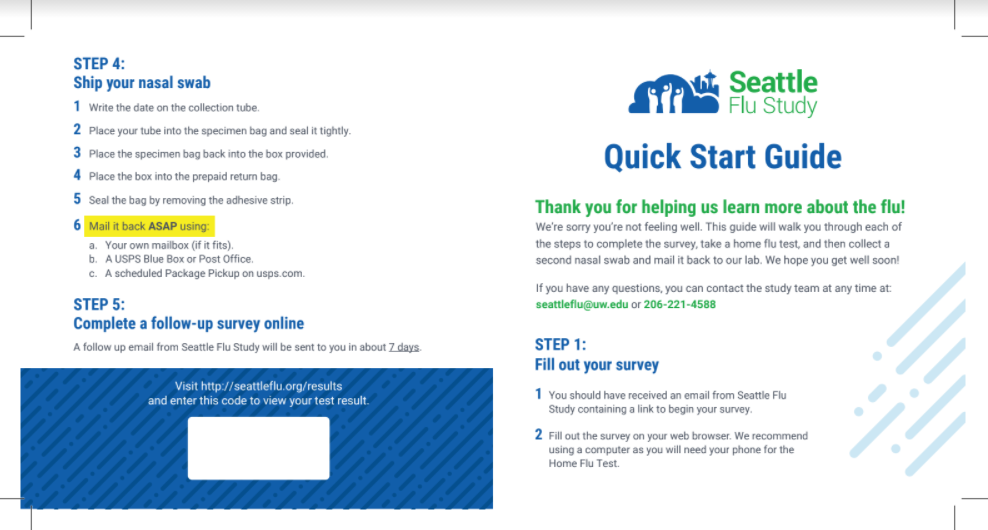


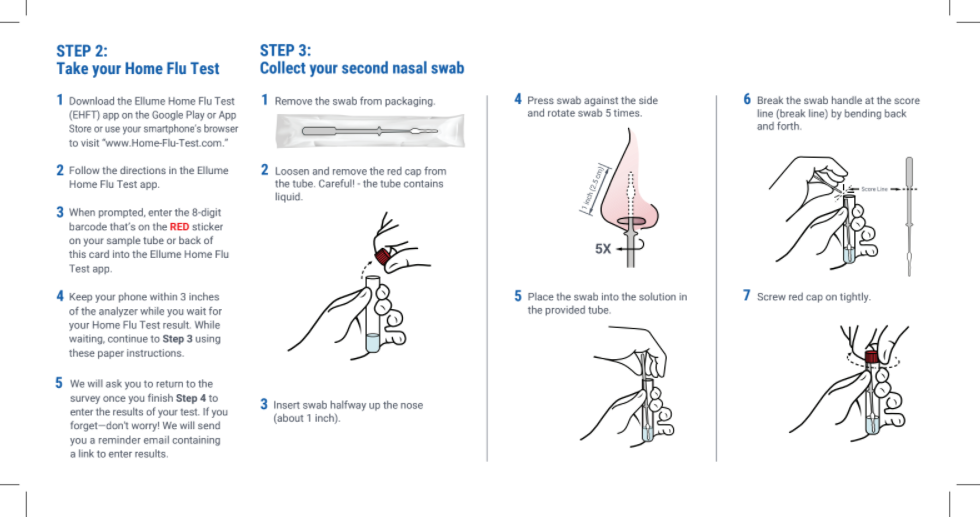

Supplement: Multimedia Appendix 1 [file publichealth_v8i2e28268_app1.docx]
