# Supplementary material for: Diagnostic Accuracy of an At-Home, Rapid Self-test for Influenza: Prospective Comparative Accuracy Study
Source: JMIR Public Health Surveill. 2022 Feb 22;8(2):e28268. doi: 10.2196/28268 (PMC8905479; doi:10.2196/28268)
Supplement: Multimedia Appendix 2 [file publichealth_v8i2e28268_app2.docx]

# Multimedia Appendix 2

## Additional participant demographics

Table S1. Counts of viral and bacterial pathogens detected in reference samples where influenza was present and absent

|  | **Total** | **Count of Influenza PCR positive** | **Count of Influenza PCR negative** |
| --- | --- | --- | --- |
| Rhinovirus | 77 | 4 | 73 |
| Influenza A | 70 | 70 | 0 |
| Human coronavirus | 50 | 0 | 50 |
| *Streptococcus pneumoniae* | 18 | 5 | 13 |
| Influenza B | 17 | 17 | 0 |
| Human metapneumovirus | 13 | 1 | 12 |
| Adenovirus | 9 | 0 | 9 |
| RSV A | 6 | 1 | 5 |
| Human parainfluenza | 4 | 0 | 4 |
| Enterovirus | 3 | 0 | 3 |
| RSV B | 3 | 0 | 3 |
| *Mycoplasma pneumoniae* | 2 | 0 | 2 |
| Enterovirus D 68 | 1 | 0 | 1 |

Table S2. Study leg time intervals, from symptom onset to taking the EHFT

| Delivery Leg | Minimum | Q1 | Mean | Median | Q3 | Maximum |
| --- | --- | --- | --- | --- | --- | --- |
| Symptom onset to study enrollment | 12 hours | 36 hours | 47.9 hours | 48 hours | 72 hours | 72 hours |
| Enrollment to Flu Kit shipped to participant | 4.2 minutes | 19.2 minutes | 6.7 hours | 1.3 hours | 11.1 hours | 98.9 hours* |
| Kit shipped to taking EHFT | .17 days | 3.0 hours | 15 hours | 5.3 hours | 16.5 hours | 13.5 days |
| Total time from symptom onset to EHFT | 14.8 hours | 46.4 hours | 62.9 hours | 69.7 hours | 85.2 hours | 346.2 hours |

*3 cases had delays longer than 90 hours and may have been caused by incorrect shipping addresses that required the operations team to follow up with the participant; 5 had delays longer 48-90 hours which could have been either incorrect addresses or delays due to a surge in kit demand that exceeded our shipping capacity
