# Supplementary material for: Diagnostic Accuracy of an At-Home, Rapid Self-test for Influenza: Prospective Comparative Accuracy Study
Source: JMIR Public Health Surveill. 2022 Feb 22;8(2):e28268. doi: 10.2196/28268 (PMC8905479; doi:10.2196/28268)
Supplement: Multimedia Appendix 3 [file publichealth_v8i2e28268_app3.docx]

# Multimedia Appendix 3

## Additional details on RDT accuracy

Table S1. Diagnostic accuracy and illness severity

*Table S1a:* Diagnostic accuracy of the RDT compared to the reference sample by number of symptoms

| **Number of self reported ARI symptoms*** | **Total influenza positive**  **N (%)** | **TP** | **FP** | **FN** | **TN** | **Sensitivity**  **(95%CI)** | **Specificity**  **(95%CI)** | **PPV**  **(95%CI)** | **NPV**  **(95%CI)** | **Influenza Crt**  **Mean**  **(SD)** | **Impact on activities (1-5)**  **Median**** |
| --- | --- | --- | --- | --- | --- | --- | --- | --- | --- | --- | --- |
| **0**  N= 18 | 2  (14.3%) | 0 | 1 | 2 | 15 | 0%  (0-84) | 94%  (62-100) | 0%  (0-97) | 88%  (64-99) | 21.6  (5.9) | 1 |
| **1**  N= 18 | 2  (11%) | 1 | 0 | 1 | 16 | 50%  (1-99) | 100%  (79-100) | 100%  (2-100) | 94%  (71-100) | 25.1  (0.7) | 2 |
| **2**  N= 60 | 2  (3.3%) | 1 | 1 | 1 | 57 | 50%  (1-99) | 98%  (91-100) | 50%  (1-99) | 98%  (91-100) | 22.0  (7.1) | 2 |
| **3**  N= 86 | 3  (3.5%) | 2 | 2 | 1 | 81 | 67%  (9-99) | 98%  (92-100) | 50%  (7-93) | 99%  (93-100) | 16.6  (3.9) | 2 |
| **4**  N= 107 | 7  (6.5%) | 6 | 4 | 1 | 96 | 86%  (42-100) | 96%  (90-99) | 60%  (26-88) | 99%  (95-100) | 17.8  (6.5) | 3 |
| **5**  N= 106 | 14  (13%) | 6 | 1 | 8 | 91 | 43%  (18-71) | 99%  (94-100) | 86%  (42-100) | 92%  (85-100) | 20.7  (3.5) | 3 |
| **6**  N= 90 | 12  (13%) | 9 | 7 | 3 | 71 | 75%  (43-95) | 91%  (82-96) | 56%  (30-80) | 96%  (82-96) | 17.8  (4.4) | 4 |
| **7**  N= 68 | 25  (37%) | 14 | 7 | 11 | 36 | 56%  (35-76) | 84%  (69-93) | 67%  (43-85) | 77%  (62-88) | 18.4  (4.2) | 4 |
| **8**  N= 34 | 15  (44%) | 11 | 1 | 4 | 18 | 73%  (45-92) | 95%  (74-100) | 92%  (62-100) | 82%  (60-95) | 18.1  (4.1) | 4 |
| **9**  N= 7 | 4  (57%) | 3 | 0 | 1 | 3 | 75%  (19-99) | 100%  (29-100) | 100%  (29-100) | 75%  (19-99) | 16.5  (3.1) | 5 |
| **No Answer**  N = 11 | 1  (6.6%) | 0 | 1 | 1 | 9 | 0%  (0-97) | 90%  (55-100) | 0%  (0-97) | 90%  (55-100) | 22.7  (N/A) | 5 |

*only includes 9 ARI symptoms out of 11 total symptoms (excluded chills, sweats, and symptoms that were asked only of children). 11 individuals did not complete the symptom survey, including 1 person positive for influenza.

**17 individuals did not complete the "impact on daily activities" question

*Table S1b:* Diagnostic accuracy of the RDT compared to the reference sample by impact on regular activities

| **How much has your current illness affected your regular activities? (score of 1-5)** | **Flu Pos**  **N (%)** | **TP** | **FP** | **FN** | **TN** | **Sensitivity**  (95%CI) | **Specificity**  (95%CI) | **PPV**  (95%CI) | **NPV**  **(95%CI)** | **Mean flu Crt** | **Mean Symptom Duration (days)** | **Median # reported symptoms** |
| --- | --- | --- | --- | --- | --- | --- | --- | --- | --- | --- | --- | --- |
| **Not at all (1)**  N= 61 | 3  (5.2%) | 3 | 1 | 0 | 57 | 100%  (29-100) | 98%  (90-100) | 75%  (19-99) | 100%  (93-100) | 15.2  (2.1) | 2.98 | 3 |
| **A little bit (2)**  N= 149 | 2  (1.4%) | 1 | 7 | 1 | 140 | 50%  (10-99) | 95%  (90-98) | 12%  (0-53) | 99%  (96-100) | 17.4  (5.2) | 2.93 | 3 |
| **Somewhat (3)**  N= 148 | 8  (5.4%) | 6 | 5 | 2 | 135 | 75%  (35-97) | 96%  (92-99) | 55%  (23-83) | 99%  (95-100) | 16.6  (3.8) | 2.76 | 5 |
| **Quite a bit (4)**  N= 116 | 22  (18.9%) | 14 | 4 | 8 | 90 | 64%  (41-83) | 96%  (89-99) | 78%  (52-94) | 92%  (85-96) | 18.8  (5.5) | 2.73 | 5 |
| **Very much (5)**  N= 114 | 50  (45.0%) | 29 | 7 | 21 | 57 | 59%  (44-73) | 90%  (79-96) | 83%  (66-93) | 73%  (61-83) | 19.3  (3.9) | 2.87 | 6 |
| **No Answer**  N = 17 | 2  (11.8%) | 0 | 1 | 2 | 14 | 0%  (0-84) | 93% (68-100) | 0%  (0-97) | 88%  (62-98) | 21.6 | 3.9 | 0 |
| **Mean**  **(SD)** | 4.3  (1.0) | 4.3  (1.1) | 3.30  (1.3) | 4.5  (0.77) | 2.9  (1.2) | n/a | n/a | n/a | n/a | n/a | n/a | n/a |

*Table S1c:* Sub-analysis mean values, grouped by PCR result and TP, FP, FN, and TN EHFT results

| **Category** | **PCR Positive for Influenza** | **PCR Negative for Influenza** | **TP** | **FP** | **FN** | **TN** | ***P* value of**  **PCR + vs. PCR-** |
| --- | --- | --- | --- | --- | --- | --- | --- |
| **Mean Symptom Duration (Days)** | **2.7** | **2.9** | 2.7 | 2.9 | 2.6 | 3.0 | .07 |
| **Mean # of ARI Symptoms** | **6** | **4.3** | 7.2 | 6.3 | 6.6 | 5.3 | < .001 |
| **Mean level of disruption to activities (1-5)*** | **4.3** | **2.9** | 4.3 | 3.3 | 4.5 | 2.9 | < .001 |
| **Mean Influenza Crt**** | **18.8** | **n/a** | 16.8 | n/a | 22 | n/a | n/a |

*5 Likert scale 1 = no disruption, 5 = most disruption

**Only includes influenza positive individuals

Table S2. False Positives and False Negatives by symptom duration and flu subtype

**False Positives**

| **Symptom Duration (Days)** | **Total False Positives** | **Influenza A False Positives** | **Influenza B False Positives** |
| --- | --- | --- | --- |
| **2** | 10 | 1 | 9 |
| **3** | 7 | 0 | 7 |
| **4** | 8 | 2 | 6 |
| **5** | 0 | 0 | 0 |
| **6** | 0 | 0 | 0 |
| **Total** | 25 | 3 | 22 |

**False Negatives**

| **Symptom Duration (Days)** | **Total False Negatives** | **Influenza A False Negatives** | **Influenza B False Negatives** |
| --- | --- | --- | --- |
| **2** | 9 | 7 | 2 |
| **3** | 10 | 8 | 2 |
| **4** | 12 | 11 | 1 |
| **5** | 3 | 2 | 1 |
| **6** | 0 | 0 | 0 |
| **Total** | 34 | 28 | 6 |
