# Supplementary material for: Diagnostic Accuracy of an At-Home, Rapid Self-test for Influenza: Prospective Comparative Accuracy Study
Source: JMIR Public Health Surveill. 2022 Feb 22;8(2):e28268. doi: 10.2196/28268 (PMC8905479; doi:10.2196/28268)
Supplement: Multimedia Appendix 4 [file publichealth_v8i2e28268_app4.docx]

# Multimedia Appendix 4

## Regression adjusted mean influenza Crt

Table S1. Regression adjusted mean influenza Crt values of influenza positive reference samples

| Measure (N = influenza positive participants) | Raw Means (SD) | Adjusted Means  (95% CI) | *P* value |
| --- | --- | --- | --- |
| Days from Symptom Onset* |  |  | .007 |
| 0* (0) | n/a | **15.5** (12.7-18.2) |  |
| 1 (0) | n/a | **16.8** (15.9-17.8) |  |
| 2 (22) | **17.1** (4.2) | **18.2** (16.3-20.1) |  |
| 3 (29) | **18.5** (4.5) | **19.52** (16.7-22.4) |  |
| 4 (28) | **19.8** (4.1) | **20.9** (17.1-24.7) |  |
| 5 (7) | **21.3** (4.8) | **22.2** (17.5-27) |  |
| 6 (1) | **22.9** (n/a) | **23.54** (17.9-29.3) |  |
| >6 (0) | n/a | **24.6** (18.3-31.6) |  |
| Number of reported symptoms** |  |  | .03 |
| 0 (2) | **21.6** (5.9) | **18.59** (14.5-22.7) |  |
| 1 (2) | **25.1** ( .7) | **18.1** (17.7-18.5) |  |
| 2 (2) | **22.0** (7.1) | **17.6** (16.8-18.5) |  |
| 3 (3) | **16.6** (3.9) | **17.1** (15.9-18.4) |  |
| 4 (7) | **17.8** (6.5) | **16.6** (15.0-18.4) |  |
| 5 (14) | **20.7** (3.5) | **16.1** (14.1-18.3) |  |
| 6 (12) | **17.8** (4.4) | **15.7** (13.2-18.3) |  |
| 7 (25) | **18.4** (4.2) | **15.2** (12.3-18.2) |  |
| 8 (15) | **18.1** (4.1) | **14.7** (11.4-18.2) |  |
| 9 (4) | **16.5** (3.1) | **14.2** (10.5-18.1) |  |
| Reported Impact on Daily Activities*** |  |  | n/a |
| Not at all (3) | **15.2** (2.1) | **12.5** (6.6-18.4) |  |
| A little bit (2) | **17.4** (5.2) | **13.7** (6.2-21.5) |  |
| Somewhat (8) | **16.6** (3.8) | **14.1** (8.3-19.8) |  |
| Quite a bit (22) | **18.8** (5.5) | **15.9** (10.6-21.2) |  |
| Very much (49) | **19.3** (3.9) | **15.8** (10.7-20.9) |  |

*Model adjusted for age;

**value of 0 included to indicate y intercept when absent from data;

***Model controlled for age and symptom duration; 5 Likert scale: 1 = not at all, 5 = very much; 3 participants missing activities data
