# Supplementary material for: Diagnostic Accuracy of an At-Home, Rapid Self-test for Influenza: Prospective Comparative Accuracy Study
Source: JMIR Public Health Surveill. 2022 Feb 22;8(2):e28268. doi: 10.2196/28268 (PMC8905479; doi:10.2196/28268)
Supplement: Multimedia Appendix 5 [file publichealth_v8i2e28268_app5.docx]

# Multimedia Appendix 5

## User experience with study procedures and specimen quality

Table S1a. **Participant reported responses to test comfort and confidence in conducting testing procedures**

| **How confident are you that you correctly completed your nasal swab?*** | **N (%)**  **N = 560** |
| --- | --- |
| Not confident | 8 (1.4) |
| Somewhat confident | 216 (38.5) |
| Very confident | 337 (60.1) |
| **Did you experience any discomfort while completing the nasal swab?***** |  |
| No discomfort | 41 (7.3) |
| Mild discomfort | 431 (76.8) |
| Strong discomfort | 89 (15.9) |
| **How confident are you that you correctly completed your Home Flu Test nasal swab?**** | **N = 567** |
| Not confident | 9 (1.6) |
| Somewhat confident | 188 (33.5) |
| Very confident | 371 (66.1) |
| **Did you experience any discomfort while completing the Home Flu Test nasal swab?**** |  |
| No discomfort | 35 (6.2) |
| Mild discomfort | 427 (76.1) |
| Strong discomfort | 106 (18.9) |

*45 missing for reference sample

**38 missing for Ellume Home Flu Test

Table S1b**,** Documented errors in returned reference samples

| Shipper box missing | 17 |
| --- | --- |
| Shipper box damaged | 0 |
| Shipper box not closed | 0 |
| Sample card missing | 0 |
| Address label wrong | 0 |
| Specimen bag not sealed | 4 |
| Specimen bag missing | 0 |
| Other | 1 |
| UTM label not filled out | 25 |
| UTM tube missing | 0 |
| UTM tube not in the specimen bag | 0 |
| UTM tube damaged | 0 |
| Swab not in UTM | 0 |
| 2 or more swabs in UTM | 0 |
| UTM leaked | 0 |
| Other | 2 |

Table S2a**:** Median Rnase P Crt values and comparisons between demographic and accuracy subgroups

| Group (N) | RNase P Crt Raw Median (SD) | *P* value |
| --- | --- | --- |
| Reference Test Result |  | .05 |
| PCR positive (87) | 22.7 (3.98) |  |
| PCR negative (518) | 20.7 (4.33) |  |
| Influenza subtype |  | .97 |
| Influenza A (70) | 22.7 (3.91) |  |
| Influenza B (17) | 22.9 (4.34) |  |
| Child Vs. Adult* |  | .34 |
| 5-17 years (33) | 20.0 (4.44) |  |
| 18+ years (572) | 21.2 (4.27) |  |
| Age* |  | .22 |
| 5-12 years (29) | 22.4 (4.47) |  |
| 13-18 years (4) | 16.6 (3.74) |  |
| 19-24 years (54) | 18.6 (4.29) |  |
| 25-34 years (222) | 21.2 (4.46) |  |
| 35-44 years (159) | 21.5 (4.12) |  |
| 45-64 years (112) | 22.1 (4.17) |  |
| >65 years (12) | 19.9 (3.13) |  |
| Test Result Subgroup |  | .02** |
| True Positive (53) | 22.5 (4.01) |  |
| False Positive (25) | 22.9 (3.24) |  |
| False Negative (34) | 23.3 (4.00) |  |
| True Negative (493) | 20.5 (4.36) |  |

*13 participants missing age data

**see Appendix 8B for post-hoc test results

Table S2b**: Dunn multiple**  **comparisons of median Rnase P Crt by test result subgroups**

| Comparison group | unadjusted p | adjusted *P* value (Holm-Bonferroni correction) |
| --- | --- | --- |
| FN vs. FP | .72 | .72 |
| FN vs. TN | .037 | .18 |
| FP vs. TN | .023 | .14 |
| FN vs. TP | .37 | .74 |
| FP vs. TP | .22 | .91 |
| TN vs TP | .23 | .70 |
